# Does schooling protect sexual health? The association between three measures of education and STIs among adolescents in Malawi

**DOI:** 10.1080/00324728.2019.1656282

**Published:** 2019-10-17

**Authors:** Barbara S. Mensch, Monica J. Grant, Erica Soler-Hampejsek, Christine A. Kelly, Satvika Chalasani, Paul C. Hewett

**Affiliations:** 1 Population Council; 2 University of Wisconsin-Madison; 3 Independent consultant; 4 London School of Hygiene and Tropical Medicine; 5 United Nations Population Fund (UNFPA)

**Keywords:** HIV, HSV-2, sexually transmitted infections, school enrolment, education, grade attainment, literacy, numeracy, adolescents, Malawi

## Abstract

While multiple studies have documented shifting educational gradients in HIV prevalence, less attention has been given to the effect of school participation and academic skills on infection during adolescence. Using the Malawi Schooling and Adolescent Study, a longitudinal survey that followed 2,649 young people aged 14–17 at baseline from 2007 to 2013, we estimate the effect of three education variables: school enrolment, grade attainment, and academic skills—numeracy and Chichewa literacy—on herpes simplex virus type 2 (HSV-2) and HIV incidence using interval-censored survival analysis. We find that grade attainment is significantly associated with lower rates of both HSV-2 and HIV among girls, and is negatively associated with HSV-2 but not HIV among boys. School enrolment and academic skills are not significantly associated with sexually transmitted infections (STIs) for boys or girls in our final models. Efforts to encourage school progression in high-prevalence settings in sub-Saharan Africa could well reduce, or at least postpone, acquisition of STIs.

## Introduction

Over the last few decades, educational participation has expanded considerably in sub-Saharan Africa (African Development Bank Group 2011, 2014; UNESCO 2011). Increases in grade attainment and schooling duration have been linked to changes in the HIV epidemic. Multiple studies have examined educational gradients in HIV prevalence among adults and how they have shifted over time (de Walque et al. 2005; Fortson 2008; Hargreaves and Howe 2010; Iorio and Santaeulalia-Llopis 2016). Studies have also investigated whether there is an association between adolescent school participation and both HIV and herpes simplex virus type 2 (HSV-2) infection (see, e.g., Hargreaves, Bonell, et al. 2008; Doyle et al. 2013; Santelli et al. 2015; Mee et al. 2018). Although these studies have established the association between both HIV and HSV-2 infection and schooling, none of them have attempted to identify which dimensions of schooling may be most salient for sexually transmitted infection (STI) prevention nor have they considered how changes in adolescent educational characteristics may be associated with the incidence of new infections across the transition to adulthood.

In order to better understand the potential protective effects of schooling for sexual health, we examine three dimensions of education—school enrolment, grade attainment, and academic skills— and their association with the incidence of HIV and HSV-2 infection among a cohort of adolescents in southern Malawi. HSV-2, commonly referred to as genital herpes, is almost always sexually transmitted and is thus considered a reliable marker of sexual behaviour among adolescents (Wagner et al. 1994; Obasi et al. 1999; Smith and Robinson 2002). Furthermore, HSV-2 infection increases the risk of HIV transmission even among those who are asymptomatic (O’Farrell 1999; Todd et al. 2006; Glynn et al. 2008; Reynolds 2009; Celum et al. 2010). Maternal HSV-2 infection also increases the risk of neonatal morbidity and mortality due to exposure in the genital tract during childbirth (Corey and Wald 2009). While many of those seropositive for HSV-2 never experience a clinical outbreak, prevention of infection has important consequences for public health, particularly in low-income countries where suppressive therapy is not readily available.

We use data from the Malawi Schooling and Adolescent Study (MSAS) to estimate the association between adolescent schooling and subsequent infection with HIV and with HSV-2. The MSAS is a longitudinal survey of adolescents that first interviewed respondents in 2007 and included HIV and HSV-2 testing in the fourth, fifth, and sixth rounds of fieldwork conducted in 2010, 2011, and 2013. Biomarkers, such as the indicators of STI status used in this analysis, provide a more objective tool than self-reports for examining whether adolescent schooling is associated with sexual risk behaviour (Mensch et al. 2003, 2008; Nnko et al. 2004; Minnis et al. 2009; Lindstrom et al. 2010; Langhaug et al. 2011; Luke et al. 2011; Kelly et al. 2013; Kelly, Chalasani, et al. 2014; Kelly, Hewett, et al. 2014). Not only are unmarried adolescent girls often reluctant to admit to being sexually active, analyses of longitudinal data from Malawi indicate that schoolgoing girls are more likely to report sexual activity inconsistently at baseline and to retract reports of ever having had sexual intercourse across rounds than their peers who have left school (Soler-Hampejsek et al. 2013). These patterns suggest that self-report data are particularly problematic for analyses of links between sexual behaviour and school enrolment.

Identifying the distinct contributions of school enrolment, grade attainment, and academic skills is important in Malawi and other sub-Saharan African countries, given late school entry, grade repetition, and intermittent dropout. These schooling processes result in considerable variability in grade attainment among students of the same age and in the number of years taken to complete any grade. In addition, we consider whether academic skills, specifically literacy and numeracy, are associated with STIs. While academic skills are significantly correlated with self-reported health (Smith-Greenaway 2015), the expansion of school enrolment and grade attainment in sub-Saharan Africa has been associated with poor learning (Kremer et al. 2013; Pritchett 2013). By disentangling the patterns of association between both HIV and HSV-2 infection and school enrolment, grade attainment, and academic skills, our study offers a more precise understanding of the potential protective effects of schooling and will inform interventions to reduce STIs among adolescents in sub-Saharan Africa.

### Pathways linking education to sexual health of adolescents

The younger an individual is when HIV is acquired, the greater the potential to transmit infection (Bekker et al. 2015). Because access to testing and treatment is considerably lower among young people, viral suppression is less likely, with consequences for the control of the epidemic (Wong et al. 2017), especially in sub-Saharan Africa where three out of four new infections are among girls aged 15–19 (UNAIDS 2018). While overall AIDS-related deaths globally have declined by over 50 per cent since 2004, among adolescents they have *increased* by a similar percentage (UNAIDS 2016, 2018).

There are a number of ways that young people can reduce the risk of STIs. They can, of course, abstain from sexual activity. If sexually active, they can use condoms, reduce coital frequency, remain monogamous, and attempt to select lower-risk partners—for example, partners who are close to them in age rather than considerably older, and partners who themselves remain monogamous. Much of the literature on the mechanisms underlying the association between education and sexual health has focused on how increased grade attainment boosts the likelihood of engaging in these protective behaviours via enhancement of cognitive, social, and material assets.

Several theoretical pathways, some protective and others enhancing risk, link school attendance —as distinct from grade attainment and skills—to infection (Jukes et al. 2008). School attendance is said to reduce the amount of time available for students to engage in sexual activity (Black et al. 2008). In addition, students’ sexual networks are said to be safer than those of adolescents who are not attending school (Jukes et al. 2008). Social norms that frame sexual activity and schooling as incompatible for girls, referred to as the ‘cultural antimony between sex and schooling’ (Frye 2017), may delay sexual debut and discourage sexual activity (Clark and Mathur 2012; Alsan and Cutler 2013). Using indepth interviews, Frye describes a shared narrative linking sexual activity with academic performance, absenteeism, and dropout among students and teachers in Malawi. Teachers are reported to punish and parents to withdraw financial support from students found to be involved in a sexual relationship, especially girls. Teachers and students describe how sexual relationships undermine girls’ concentration, tempt them to miss school, and lead to pregnancies. While there is no evidence that absenteeism and school performance among girls is actually affected by relationships with boys, the narrative shapes the behaviour of parents and teachers, and may act as a deterrent to engaging in sexual activity.

For some girls, attending school may present risks. Numerous reports have been published about widespread school-related gender-based violence (SRGBV) in low-income countries (Dunne et al. 2006; Gerver 2013; EFA Global Monitoring Report et al. 2015; Psaki et al. 2017). Abuse of girls has reportedly been perpetrated by male classmates as well as by male teachers, who may exchange sexual intercourse for better grades. Such abuse is said to have serious consequences for emotional and physical health including increasing risk of STIs (Management Systems International 2008), although empirical support for SRGBV’s effect on health outcomes is lacking (Psaki et al. 2017). There is also evidence, often anecdotal, that girls exchange sexual favours for school fees or other financial support in order to remain in school (Luke 2003).

Whether school potentially exposes students to risk may vary by student performance and local context. In a longitudinal survey of secondary school students in Lilongwe District, Malawi, girls attending schools with more grade repetition had a higher likelihood of initiating sexual activity between two survey waves, controlling for a variety of individual-level and other school-related factors (Kim 2015). A study in Cape Town that explored the effect of individual repetition rather than school-level repetition found that students who were attending the appropriate grade for age were more likely to have initiated sexual activity than their female peers who had repeated grades, potentially due to their exposure to male students two to three years older, who may be more interested in engaging in sexual activity (Lam et al. 2013). Schooling discontinuities, including grade repetition and temporary withdrawals from school, have also been linked to a higher risk of schoolgirl pregnancy (Grant and Hallman 2008).

### The association between educational attainment and HIV

Demographers have long observed that the better educated are healthier than their less educated counterparts (Caldwell 1979; Cochrane et al. 1980; Bledsoe et al. 1999). An extensive literature has documented a strong association between educational attainment and reduced fertility as well as lower maternal, infant, and child mortality (Mensch et al. 1985; Cleland and van Ginneken 1988; Jejeebhoy 1995; Gakidou et al. 2010; Pamuk et al. 2011; Bhalotra and Clarke 2013; Lutz and Kebede 2018). Time spent in school has been expected to influence each of these outcomes via developing cognitive skills, challenging traditional beliefs about disease and folk remedies, and transmitting knowledge about behaviours that can promote health. In addition to these pathways, education is thought to develop women’s autonomy by improving their status within the family, enabling them to act on health knowledge, and increasing their utilization of health services (Caldwell 1979; Jejeebhoy 1995). Education is also said to alter fertility preferences and increase the opportunity cost of childbearing for women (Caldwell 1980). Microeconomic theory posits that individuals who have invested in their education have more of an incentive to protect their health because of greater expected returns in the future (Becker 1993; de Walque 2004). Furthermore, microsociological theory suggests that the social networks of more educated individuals may contain better health information and outcomes than those of less educated individuals (Montgomery and Casterline 1996).

By extrapolation, these same mechanisms should lead to lower rates of HIV infection among more educated individuals. Yet, since the onset of the epidemic, the direction and strength of the association between educational attainment and HIV in sub-Saharan Africa has varied across space and time (Hargreaves and Glynn 2002; de Walque 2004; Kum-wenda et al. 2006; Barnighausen et al. 2007; Fortson 2008). Beginning in the early 1990s, research on the link between educational attainment and HIV in sub-Saharan Africa indicated that HIV prevalence was higher among those with more years of schooling (Dallabetta et al. 1993; Fylkesnes et al. 1997; Smith et al. 1999; Hargreaves and Glynn 2002). These studies found that those with more schooling were more likely to live in urban areas, were more mobile, and had greater disposable income, characteristics associated with risk behaviours such as having more (and more heterogeneous) sexual partners, extramarital relationships, and delayed marriage. As the epidemic matured and information disseminated about the cause of HIV and the ways to reduce risk, the positive gradient between educational attainment and HIV found in many countries early in the epidemic disappeared or reversed in many settings (de Walque et al. 2005; Hargreaves, Bonell, et al. 2008). Baker et al. (2017) have argued that the positive or shifting education gradient with certain risky health behaviours or diseases including HIV in sub-Saharan Africa is not counter-intuitive but is consistent with a conceptualization of the relationship between educational attainment and health as having multiple and sometimes offsetting pathways. Indeed, Gregson et al. (2001) observed that while more educated populations were particularly vulnerable at the early stages of the epidemic, they were also better able to respond effectively.

These earlier studies investigated the association between educational attainment and HIV among adults but did not establish a causal link. In contrast, two recent analyses of the effect of schooling on HIV status in sub-Saharan Africa have taken advantage of natural experiments to account for the potential endogeneity of schooling; both found significant negative effects of grade attainment on adult infection. An analysis of Demographic and Health Survey (DHS) data from Malawi and Uganda used the implementation of universal primary education policies to estimate the association between increased schooling and a reduction in adult HIV infection (Behrman 2015). An analysis of Botswanan data also used an educational policy reform that led to increases in educational attainment to identify a significant inverse effect of an additional year of secondary schooling on the probability of HIV infection among a sample of men and women aged 18–32 (De Neve et al. 2015).

### The association between school enrolment and STIs among adolescents

In addition to the considerable body of research on the association between educational attainment and HIV among adults, a number of studies have investigated the association of school attendance and HIV or HSV-2 among adolescents. A study conducted among a random population-based cross-sectional sample of rural South Africans aged 14–25 found that school attendance was associated with lower HIV prevalence among young men but not young women (Hargreaves, Morison, et al. 2008). A more recent study examined the associations between current attendance in post-primary schooling, sexual behaviour, and HSV-2 among a large sample of unmarried 15–24-year-olds in rural Mwanza, Tanzania. The authors found that adolescents, both boys and girls, who reported attending school (the vast majority in secondary school) were less likely to be sexually active, less likely to report multiple and concurrent partners, more likely to use condoms, and less likely to be infected with HSV-2 (Doyle et al. 2013). An even more recent analysis of DHS data from nine sub-Saharan African countries assessed the association between school enrolment and HIV status, and found evidence of a significant effect in three countries but not in six others, including Malawi (Mee et al. 2018). In addition to these cross-sectional studies, a longitudinal study in Rakai, Uganda found that HIV incidence for young women in school was one-quarter as high as for those not enrolled, but the association was not significant for men (Santelli et al. 2015). The authors suggested that school enrolment, which increased considerably during the nine survey rounds between 1999 and 2011, lowered the risk of HIV by delaying sexual initiation among female students.

While the findings from these papers are suggestive of a causal relationship between school attendance and HIV, the research has not addressed analytically the possibility that the same factors affecting school status, many of which are unobservable, also affect the likelihood of engaging in risky behaviours. Alsan and Cutler (2013) used distance to school, conditional on a set of demographic and locational controls, to address this endogeneity and found that girls’ enrolment in secondary school significantly increased the likelihood of sexual abstinence, although as already noted, it may well be that schoolgoing girls are more likely to under-report sexual activity.

Several recent randomized control trials have also examined the relationship between school attendance and HIV or HSV-2 prevalence in sub-Saharan Africa. Baird et al. (2012) examined the effect of a cash transfer programme for schooling in Malawi on HIV and HSV-2 prevalence among young unmarried women. The cash transfer group was divided between a conditional group that received cash payment if school was attended for 80 per cent of the days that it was in session during the previous month, and an unconditional group to whom payment was provided regardless of attendance. Significant differences in STIs were found between the cash transfer groups and a control group that did not receive any payment, but not between the conditional and unconditional intervention groups. The findings are consistent with the assertion that poverty makes girls more vulnerable to risk behaviour, but they do not provide evidence for a protective effect of school participation on infection.

A second randomized evaluation of a conditional cash transfer intervention in South Africa has provided some direct evidence of the role of school attendance on STI outcomes (Pettifor et al. 2016). In the study, over 2,500 young women aged 13–20 and enrolled in grades 8–11 were recruited in the rural Mpumalanga province. The adolescents and their parents who were randomly assigned to the treatment group received a monthly cash transfer conditional on school attendance, with both receiving a cash payment for up to three years if attendance exceeded 80 per cent of school days. The study did not find any effect of the conditional cash transfer on HIV or HSV-2 infection, despite the finding that it did reduce the prevalence of some risky sexual behaviours and physical violence from a partner. However, results indicated a direct link between schooling attendance and HIV acquisition, with an increased relative risk of infection of 1.88 (95 per cent confidence interval: 1.08–3.27) for adolescents missing more than 20 per cent of school days, controlling for whether they received the conditional cash transfer. This finding was observed despite attendance being consistently high (95 per cent) for young women in both the intervention and control groups.

Finally, Duflo et al. (2015) conducted a seven-year randomized evaluation of the effect of an education subsidy to sixth graders in Kenya on HIV and HSV-2, although HIV prevalence was too low to assess in the analysis. The subsidy was in the form of the provision of free school uniforms to upper primary school students. They found that students who received free uniforms were significantly less likely to drop out before the end of primary school, but this did not have any significant effect on HSV-2 acquisition. However, students who received the free uniforms in combination with HIV education were significantly less likely to be infected with HSV-2 seven years later. This study, however, did not disentangle the effects of prolonged school enrolment from increased grade attainment.

### Study context

#### Education in Malawi

Although Malawi ranks at the low end among African countries in grade attainment, the elimination of primary school fees in 1994 has enabled the country to achieve nearly universal access to primary school (Al-Samarrai and Zaman 2007). In 2010, only 4 per cent of men and 8 per cent of women aged 20–24 had never attended school compared with 11 per cent of men and 34 per cent of women aged 40–44. Moreover, 51 per cent of men and 38 per cent of women aged 20–24 had completed the eight standards (grades) of primary school compared with 29 per cent of men aged 40–44 and 16 per cent of women (National Statistical Office and ICF Macro 2011). Enrolment and attainment have improved further between 2010 and 2016, particularly among women (National Statistical Office and ICF Macro 2011).

Although access to school has increased, it has had little positive impact on other schooling outcomes, which is likely due to stagnant or declining school quality. As primary school gross enrolment rates increased from 131 to 146 between 2008 and 2015, the number of students per class rose from 85 to 126 (World Bank 2018). According to the results of numeracy tests administered in Standard ‘6’ in Malawi, no students scored in the ‘competent’ range or above (UNESCO 2005). Indeed, Malawi reported the lowest mean literacy score and second to lowest numeracy score of 14 countries in the third round (2006–11) of the Southern and Eastern Africa Consortium for Monitoring Educational Quality assessment (SACMEQ 2018). Although policies and interventions to encourage girls’ school enrolment have contributed to near parity between boys and girls in primary school entry (Chimombo et al. 2000; Anzar et al. 2004), girls are still more likely to leave school at a younger age than boys (Baird et al. 2012). Moreover, due to late entry, repetition, and temporary withdrawal, there is considerable variation in grade attainment among students of the same age (Sunny et al. 2017).

#### HIV and HSV-2 prevalence in Malawi

According to the 2015–16 DHS, HIV prevalence in Malawi in adults aged 15–49 was estimated to be 8.8 per cent: 10.8 per cent among women and 6.4 per cent among men. Among those aged 20–24, prevalence was estimated to be 6.4 and 4.0 per cent among women and men, respectively. There is substantial geographic variability; in the Southern region, where our baseline survey was conducted, overall prevalence was estimated to be 12.8 per cent, substantially higher than that for the country as a whole (National Statistical Office and ICF Macro 2011). The DHS data indicate a U-shaped association between years of schooling and HIV prevalence for men but not for women: men with no schooling and those with some secondary education are both more likely to be infected than those who only attended primary (National Statistical Office and ICF Macro 2011). The data also indicate a positive wealth gradient in HIV prevalence among both men and women (National Statistical Office and ICF Macro 2011). Considerable variability in prevalence is also observed among ethnic groups, with differences in HIV prevalence speculated to be related to differences in sexual practices that elevate risk (Poulin and Muula 2011).

Less is known about HSV-2 in Malawi than about HIV, as HSV-2 testing is not common. However, some general patterns regarding the epidemiology of HSV-2 in sub-Saharan Africa are worth noting. There is a multifold difference in the prevalence in HSV-2 compared with HIV (Glynn et al. 2014). Like HIV, prevalence of HSV-2 is higher among young women than among young men, both because women’s partners are typically older and because adolescent girls are more susceptible to infection due to cervical ectopy (Smith and Robinson 2002). According to studies in rural northern Malawi, HSV-2 prevalence increases steeply with age and then typically plateaus later in adulthood (Glynn et al. 2008).

## Methods

### Data

The MSAS is a six-round longitudinal study of 2,649 adolescents who were resident in two contiguous rural districts in the Southern region of the country and reported to be aged 14–16 in January 2007. The initial 2007 sample consisted of 1,764 students (875 girls and 889 boys) who were randomly selected from the enrolment rosters at 59 randomly selected primary schools in Machinga and Balaka Districts. Because class registers are often missing, we distributed new registers in all sample schools at the beginning of the school year. To account for potential age misreporting in the registers, all enrolled students aged 13–18 were randomly sampled from the registers. No one at the school was informed about the eligibility criteria. The probability of a particular school being included was proportional to its enrolment in 2006. At each school, approximately 30 students stratified by age and sex who were enrolled in Standards ‘4’ to ‘8’ (the last five grades of primary school) were interviewed. An additional sample of 885 adolescents (462 girls and 423 boys) not enrolled in school was drawn from the communities surrounding the selected primary schools. These respondents, referred to as the ‘out-of-school’ sample because of their status when first interviewed, were identified through key informants located at the school or in the randomly selected school catchment villages. The study’s ratio of 14–16-year-olds attending Standards ‘4’ to ‘8’ relative to those out of school was dictated by the proportion observed in the 2004 DHS for Malawi. The final sample contained youth aged 14–17 years old, due to ageing between the start of the school year and the time of the interview. Interviews were conducted annually from 2007 (Round 1) to 2011 (Round 5) and in 2013 (Round 6). The study successfully reinterviewed 91, 90, 88, 88, and 82 per cent of the original sample in 2008, 2009, 2010, 2011, and 2013, respectively.

The MSAS adolescent questionnaire includes an extensive set of questions on household and family characteristics, educational attainment, schooling history and experiences, household labour and employment, health, marriage, and sexual behaviour (Population Council 2015).

Beginning at Round 4 (2010), respondents were tested for HIV and HSV-2 by enumerators trained in counselling and testing, following the Ministry of Health’s guidelines. Testing was done at home for the majority of respondents; some were tested at schools or workplaces, with considerable effort made to maintain privacy. Both HIV and HSV-2 specimens were collected via finger pricks. A serial algorithm was used for HIV rapid testing: if respondents tested positive using the Determine^®^ HIV1/2 test (Abbott, Japan), they were retested using the Uni-Gold™ Recombigen® HIV1/2 test (Trinity Biotech, Ireland); the SD Bioline HIV 1/2 3.0 test (Standard Diagnostics, South Korea) was the tiebreaking third test. The HSV-2 samples were collected in Microtainers and transported to the College of Medicine-Johns Hopkins University Research Project laboratory at Queen Elizabeth Central Hospital in Blantyre, Malawi, for testing using the Kalon ELISA HSV-2 antibody test. More details on the testing protocol are provided in the Appendix.

### Analysis

In this paper we analyse the timing of HSV-2 and HIV infections using interval-censored survival analysis. We do not have precise information about the date of infection, but observe respondents’ infection status in 2010, 2011, and 2013. The youngest respondents were 17 years old in 2010 and the oldest were 23 in 2013. Interval-censored regressions are appropriate for data where it is known that an observation occurs within a specific interval of time (*L*_*i*_, *R*_*i*_]. Here, *L*_*i*_ is the last age at which a respondent is known to be negative, *R*_*i*_ is the first age at which a respondent is known to be positive, and the survival time, *T*, is known to occur between *L*_*i*_ and *R*_*i*_, such that *L* < *T ≤ R.* This analytic approach accommodates the irregular time intervals between surveys, the range of ages over which testing occurs, and irregular testing participation, such as for respondents not tested in 2010 who were tested in subsequent survey rounds. For respondents who were HSV-2 or HIV positive the first time they were tested, the observation is left-censored (·,*R*_*i*_]. Rather than treat the left-censored intervals as undefined, we examine HSV-2 models that assume all respondents are HSV-2 negative at age 14 and HIV models that assume all respondents are HIV negative either at age 14 or later at age 16. Given that 8 per cent of 17-year-old respondents were HSV-2 positive, we do not believe that it is reasonable to assume that no respondents were infected at age 16. Therefore, we only present the results of the HSV-2 regressions where all respondents are assumed to be HSV-2 negative at age 14. In contrast, only 1.4 per cent of 17-year-old respondents were HIV positive, suggesting that most respondents would be HIV negative at age 16.

Respondents who were HSV-2 or HIV negative the last time they were tested are right-censored (*L*_*i*_-]. Respondents who were never tested—either due to testing refusal or sample attrition—and respondents whose only test results are indeterminate do not provide information to the model and are excluded from the analysis.

The models are estimated using interval-censored survival analysis that allows for time-varying covariates (Sparling et al. 2006). In this model, time-dependent covariates are updated every time the respondent provides a valid HSV-2 or HIV test. All respondents enter the analysis at *τ*_*ij0*_, the age at which all respondents are assumed to be STI negative, which is set to age 14 or 16 depending on the model. The set of update times {*τ*_*ij*_} may differ among respondents. Preliminary analyses indicated that a Weibull distribution provides the best model fit. Therefore, we follow the model developed by Sparling et al. (2006) to estimate the hazard function (*λ*) for respondent *i* at time *j* as:

$$\lambda \left(\tau_{ij}|z_{i},y_{ij}\right)=\alpha \beta_{ij}\tau_{ij}^{\alpha -1}$$

where *z*_*i*_ is a vector of time-invariant covariates and *y*_*ij*_ is a vector of time-varying covariates. Note that *β*_*ij*_ is the rate parameter conditional on the values of the covariates at each update time *τ*_*ij*_, specified as:

$$\beta_{ij}=\exp\left(\theta +\gamma^{\prime }z_{i}+\eta^{\prime }y_{ij}\right).$$

The model also includes the scale parameter *α.* When 0< *α* <1 the hazard will be decreasing, when *α* =1 the hazard will be constant, and when *α* >1 the hazard will be increasing (Sparling et al. 2006). Censoring is assumed to be non-informative: the timing of MSAS data collection is independent from the disease incidence. The likelihood function reflects the interval censoring and is estimated using both the probability that an infection occurred in a particular interval of time and the probability that the respondent survived the observation interval without an infection occurring (Leung et al. 1997; Zhang and Sun 2010). Regressions are estimated using the user-generated ‘PROCINT’ command in SAS (Sparling et al. 2006). In all analyses, the standard errors are adjusted for the clustered sampling design, with the school being the unit of clustering.

#### Explanatory variables

Our time-varying covariates (*y*_*ij*_) include school enrolment status, highest grade completed, academic skills, maternal orphanhood, and paternal orphanhood. As noted in the description of the sampling design, school enrolment at Round 1 (when respondents were aged 14–17) was determined by whether the respondent was listed on school registers. Age-specific school enrolment before the first survey round was identified from a detailed schooling history. In subsequent rounds, enrolment was determined in response to a question about whether the respondent was currently attending school. We also control for the highest grade (standard) attained at each update point. Our third schooling variable, academic skills, is obtained from an assessment conducted in conjunction with the survey. At each round respondents were considered to be literate in Chichewa, the predominant local language, if they could read two sentences aloud. The numeracy assessment consisted of twelve questions drawn from the Malawi Institute of Education achievement tests for Standard ‘3’ and included sequencing and ordering numbers, addition, subtraction, multiplication, division, and two simple word problems. Numeracy, therefore, is operationalized as the number of correct responses. Eight per cent of respondents refused to take the numeracy assessment, citing their illiteracy; these respondents are assigned a score of zero correct answers. Unlike the other time-varying covariates included in our model, literacy and numeracy skills are not observed before the first survey round and are, therefore, not available for all respondents when aged 14. Thus, we restrict the HSV-2 models that include skills to the subsample aged 14 at baseline. For HIV models that include skills, we focus on the models where respondents are assumed to be HIV negative at age 16, in order to use the full sample. Although 10 per cent of respondents were 17 years old at the time of the interview, there is no meaningful difference between the literacy and numeracy of the 16-and 17-year-olds in our sample, so we use the skills measured at age 17 as a proxy for the skills attained at age 16. Finally, we also include time-varying measures of maternal and paternal orphanhood, given that orphanhood and caregiver instability are considered to increase risk behaviour and vulnerability to HIV in sub-Saharan Africa (Birdthistle et al. 2008; Goldberg 2013).

We also include a set of time-invariant control variables (*z*_*ij*_). First, we include a four-category measure of ethnicity, as considerable variability in HIV prevalence is observed among ethnic groups in Malawi (National Statistical Office and ICF Macro 2011). We also include parental education. Finally, because the risk of acquiring an STI is partially a function of the prevalence of infection in the particular locale where a respondent resides, we include the prevalence of HSV-2 or HIV at Round 4 in the original school catchment area in the HSV-2 and HIV models, respectively. In all cases, we exclude the respondent from the calculation of the aggregated variable.

## Results

Table 1 presents the descriptive statistics for the sample. Note that almost 10 per cent of respondents had no HSV-2 test result. For those without a test result, approximately half had refused HSV-2 testing, and slightly over one-third were lost to follow-up; the remaining respondents either had indeterminate results or an insufficient blood sample was collected. We investigated whether respondents without a test result differed in any systematic way from respondents with at least one valid test. There were no observable differences for female respondents. For male respondents, there were significant ethnic differences in the odds of being tested for HSV-2.

**Figure 1:**
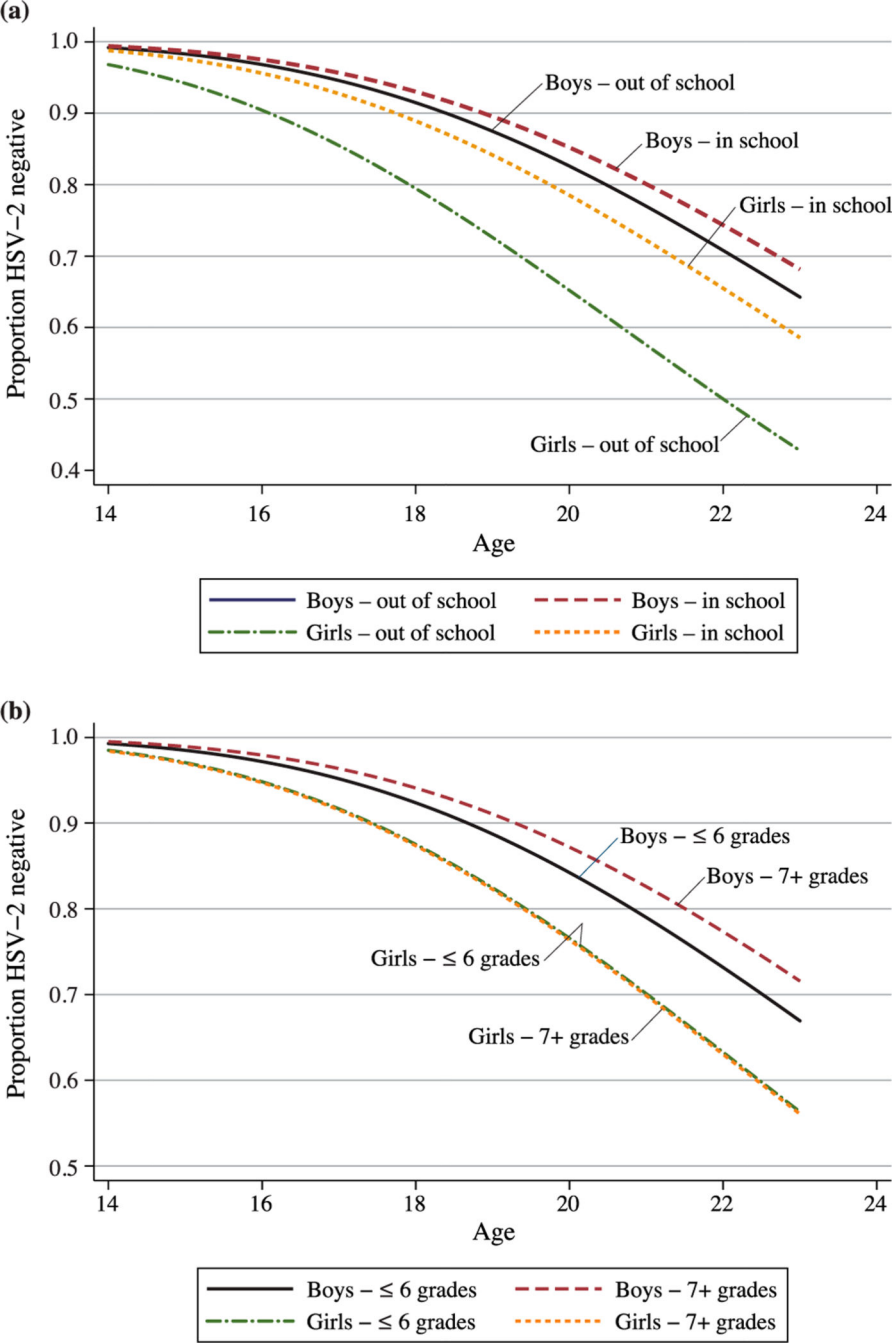
(a) HSV-2 survival curve, by sex and school enrolment status at age 14. (b) HSV-2 survival curve, by sex and grade attainment at age 14 *Note:* Based on respondents with at least one valid test result. *Source:* Authors’ analysis from MSAS data.

At the time of first testing, 13.4 per cent of respondents tested positive for HSV-2. An additional per cent of respondents seroconverted by the final survey round. Both initial infection rates and seroconversion were significantly higher for young women than for men; among respondents with at least one valid test result, 30 per cent of female respondents vs. 18 per cent of male respondents were HSV-2 positive by Round 6. Our life table estimates indicate that by age 23, we would expect more than 30 per cent of men and almost 45 per cent of women to be infected with HSV-2. Figures 1(a) and (b) present the survival curves for HSV-2 by sex and, respectively, enrolment status (in vs. out of school) and grade attainment (≤6 grades vs. 7+ grades) at age 14, based on respondents with at least one valid test result. Unadjusted for other covariates, there are significant differences in HSV-2 prevalence by enrolment status among girls but not among boys, such that girls who were enrolled in school at age 14 were less likely to be infected than girls who were out of school at that age. There is no difference by the dichotomous time-invariant measure of grade attainment for girls and the small difference for boys is not significant.

Less than 2 per cent of all respondents were HIV-positive the first time they were tested, and only 1.6 per cent of respondents seroconverted between survey rounds (Table 1). By the last round in 2013, 6.1 per cent of young women and 1.3 per cent of young men with at least one valid test result were HIV-positive. Relative to HSV-2 testing, a lower percentage of respondents had no valid HIV test result due to lower rates of testing refusal and indeterminate results. As with the HSV-2 results, there were no observable differences for female respondents with and without valid test results. For male respondents, there were significant ethnic differences in the odds of being tested for HIV, and school-level HIV prevalence in 2010 was positively associated with having at least one valid test result.

**Figure 2:**
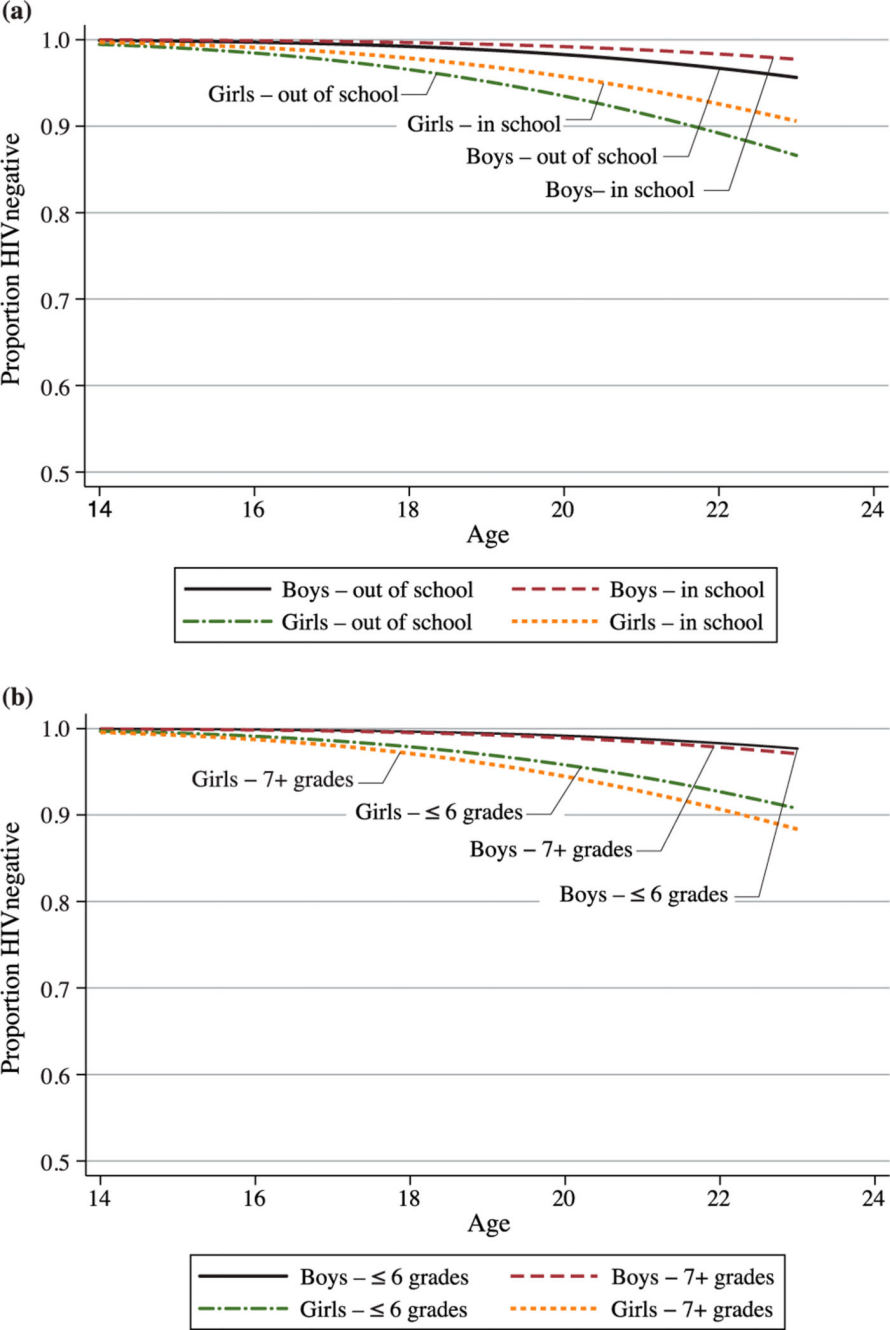
(a) HIV survival curve, by sex and school enrolment status at age 14. (b) HIV survival curve, by sex and grade attainment at age 14 *Note:* Based on respondents with at least one valid test result. *Source:* As for Figure 1.

Figures 2(a) and (b) present the survival curves for HIV by sex and by enrolment and grade attainment, respectively, based on respondents with at least one valid test result. Life table estimates indicate that by age 23, we would expect almost 2 per cent of men and more than 8 per cent of women to be infected with HIV. As with HSV-2, unadjusted for other covariates, differences in HIV by enrolment status are greater for girls than for boys, although given the much lower prevalence the differences by enrolment status as well as by grade attainment are very small and not significant.

While approximately the same percentage of boys (84 per cent) and girls (85 per cent) were enrolled in school at age 14, a gap in enrolment by sex can be observed at age 16, with 54 per cent of girls and 69 per cent of boys still enrolled (Table 1). As noted earlier, given that children enter primary school at different ages and frequently repeat grades, considerable variability is observed in grade attained by a particular age. The distribution of attainment at age 14 for the sample was as follows: 14 per cent in grades ‘1’ to ‘3’, 18 per cent in grade ‘4’, 20 per cent in grade ‘5’, 22 per cent in grade ‘6’, 18 per cent in grade ‘7’, and 8 per cent in grade ‘8’, with the average respondent completing 5.33 grades (standard deviation (SD) = 1.62). By age 16, the average respondent had gained almost one full grade since age 14, with higher variability in attainment than at age 14 (mean = 6.29; SD = 1.94). As for academic skills, out of the twelve Standard ‘3’ numeracy questions, respondents answered 7.8 correctly, on average, at age 14 and 8.1 correctly at age 16. Seventy-six per cent of the sample was considered literate in Chichewa at age 14 and 81 per cent at age 16.

Table 2 presents the results of the interval-censored survival analysis for HSV-2 infection, separately for adolescent girls and boys. The exponentiated coefficient of the independent variable can be interpreted as the hazard ratio. Although Figure 1(a) shows a significant difference for girls in the unadjusted HSV-2 survival curves by school enrolment status at age 14, girls’ school enrolment is not significantly associated with HSV-2 infection once it is allowed to vary over time and control variables are included in the model. In contrast, grade attainment is significantly associated with a lower hazard of infection among girls; each additional grade attained is associated with a 7.0 per cent lower hazard of HSV-2 infection (*β* = −0.073). Table 2 also shows that school enrolment is marginally significantly associated with the hazard of HSV-2 infection for boys, but the association is positive, such that boys have a 1.37 times higher hazard of HSV-2 infection (*β* = 0.313) if they are still enrolled in school, controlling for highest grade attained. Similar to the results for girls, each additional grade attained by boys is associated with an 8.6 per cent lower hazard of HSV-2 infection (*β* = −0.090).

Several other covariates are also associated with the timing of HSV-2 infection. There are significant differences in the timing of HSV-2 infection across ethnic groups, but not by parental education. The prevalence of HSV-2 in 2010 for the school catchment area where the participant resided in Round 1 is significantly associated with HSV-2 infection for girls; for every additional ten percentage point increase in HSV-2 prevalence, the hazard of HSV-2 infection increases 1.42 times *(β* = 0.035) for young women.

Table 3 presents the results of similar models estimated for the hazard of HIV infection, with the age at which respondents are assumed to be HIV negative set successively at ages 14 and 16. There are no significant associations between school enrolment status and the hazard of HIV infection for male or female respondents in the models with the origin set at age 14. In models assuming respondents are uninfected at age 16, there is a larger negative association between school enrolment and the hazard of HIV infection among young women, but this association is not quite statistically significant (*p* = 0.1014). Grade attainment is negatively associated with the hazard of HIV infection for girls when the origin is set at either age 14 or 16, but is not significant for boys in either model. Each additional grade of schooling attained for girls is associated with a 14.0 per cent (assuming HIV negative at age 14) or 10.6 per cent (assuming HIV negative at age 16) lower risk of HIV infection.

For adolescent boys, only orphanhood is significantly associated with the timing of infection; deaths of mothers and fathers are associated with higher hazards of HIV. The coefficients weaken and the associations become only marginally significant when the origin moves from age 14 to 16, but the differences across the models are not statistically significant. There is no significant association with orphanhood among young women. In addition, HIV prevalence in the sample school catchment area is positively associated with the hazard of infection for women.

Finally, we also estimate models that include literacy and numeracy skills (Table 4). As noted earlier, in order to assess the effect of skills on the hazard of HSV-2 we estimate a model restricted to respondents aged 14 at the first survey round. For HIV, we present only the model with the origin set at 16 in order to maximize the available sample. None of the skill categories are significantly associated with the hazard of STIs in models that included grade attainment and enrolment.

Because the correlations between our three education variables are relatively high—ranging from 0.45 to 0.62—we also performed robustness checks, estimating a series of models where we excluded one or more of the education variables to determine if that would affect our conclusions about the importance of grade attainment, especially for girls. Results from this alternate set of models are presented in the supplementary material, along with the results from the models presented in the main tables for comparison. These robustness checks did not alter our overall conclusions. The academic skills variables are never significant in models with grade attainment or enrolment. Moreover, they are only ever marginally significant (*p*-value of 0.10); Chichewa literacy is negatively associated with HIV for girls when numeracy is also excluded (see Table F in the supplementary material) and numeracy is negatively associated with HSV-2 for girls when Chichewa literacy but no other education variable is included (see Table G). While being in school during adolescence is consistently negatively associated with infection for girls, it is only significant in HIV models and only in models assuming respondents are uninfected at age 16 (see Tables A–D). For boys, depending on the model, the direction of the association between enrolment and the STI outcome varies and, as noted earlier, is significant, albeit marginally, in the model without skills where being in school raises the risk of infection; this is the only model where enrolment is significant for boys. Whereas grade attainment for girls is consistently negatively associated with infection, and significant (except for the HSV-2 model including both skills and enrolment that is limited to the reduced sample age 14 at baseline (Table C)), for boys the sign for grade attainment varies in the HSV-2 models depending on the inclusion of other variables.

## Discussion

School has been characterized as an important socializing institution, particularly for young women (Caldwell 1979; Lloyd and Mensch 1999; LeVine et al. 2012). Not only is it thought to improve longer-term health by challenging traditional norms and behaviours, it also has the potential to affect sexual behaviour for students in school. The analysis reported here, based on a longitudinal sample conducted in two southern districts in Malawi, finds evidence that the greater the grade attainment in adolescence, the less likely a girl is to be infected with HSV-2 or HIV and the less likely a boy is to be infected with HSV-2. However, no significant associations are observed between academic skills and infection in models with the full set of education variables, indicating that it is progress through school that matters most for postponing HIV and HSV-2 acquisition during the transition to adulthood. That the effect of grade attainment is not robust to different model specifications for boys for HSV-2 and never significant for HIV suggests that, in comparison to girls, it may be less important for STI prevention.

How might we interpret the effect of grade attainment, given that academic skills and enrolment in school are not as consistently associated with infection in multivariate models? Undoubtedly, adolescents who continue to progress to higher grades differ from their counterparts with lower attainment; they may be more ambitious, better behaved in school, more studious, and less likely to be absent, and may have greater educational expectations, attributes likely associated with less (risky) sexual activity, particularly for girls for whom engaging in sexual activity while enrolled in school is potentially more consequential than it is for boys (Kirby 2002; Frye 2012). Indeed, compared with boys, girls who have progressed far in school are likely to be more selective. In short, it may be the characteristics associated with higher grade attainment for girls rather than grade attainment in and of itself that reduces the likelihood of infection. According to Frye’s (2017) qualitative analysis in Malawi, becoming involved in a sexual relationship is a socially sanctioned way for girls to leave school, as it is a first step in the marriage process. Thus, girls who do not wish to marry at a young age and want to remain in school and advance to secondary are less inclined to become sexually active.

But grade attainment may not just represent a selection effect; it may also be that progression through school is a self-perpetuating process, particularly for girls who encounter competing roles at puberty. The further a student goes in school, the further she may want to go, and the more she may identify as a student rather than a young woman on the marriage market. As described by Frye (2017), sexual activity and schooling are considered incompatible for girls in Malawi. While female students are thought to enjoy the attention and gifts that a sexual relationship brings, and dream about marriage and motherhood, not all girls are vulnerable to these so-called ‘temptations’; those with greater grade attainment in adolescence may be more inclined to think of themselves as students rather than potential wives and mothers. Being a ‘schoolgirl’ and wearing a school uniform in sub-Saharan Africa is thought to signal or confer a special status, even for girls who are post-puberty (Bledsoe 1990). In addition, students and those with more education are said to have smaller, less risky sexual networks than their less educated counterparts; norms within social networks may reinforce safer behaviours for both boys and girls (Biddlecom et al. 2008; Hargreaves, Morison, et al. 2008; Jukes et al. 2008; Lloyd 2009; Handa et al. 2014). Microeconomic theory posits that individuals who invest in their education have more incentive to protect their health because of greater expected returns in the future (Becker 1993; de Walque 2007). The messages conveyed by teachers regarding the importance of staying in school and passing school-leaving exams may also affect student attitudes regarding the desirability of early sexual activity (and marriage, for girls). Finally, school participation is also thought to challenge students’ traditional notions of status hierarchies, with education and occupation replacing age and sex as factors affecting societal ranking (LeVine et al. 2001). Where such a shift in world view takes place, it may affect the willingness of students to engage in sexual activity.

Finally, that literacy and numeracy are not significant in the HSV-2 or HIV models does not mean skill acquisition is unimportant for longer-term health outcomes or that those with more education are not more knowledgeable about HIV prevention and better equipped to act on that knowledge. Those with higher grade attainment may possess greater non-cognitive skills and have acquired the interpersonal and communication competencies necessary to navigate health institutions and acquire targeted knowledge. They may also be more likely to understand ‘safe sex’ messages.

### Limitations

There are a number of limitations to the analyses presented here. First, we are unable to rule out the possibility that the association between adolescent school enrolment and HSV-2 infection is co-determined. As noted in the previous section, we cannot determine whether the effect of grade attainment is simply a selection effect or represents an underlying causal mechanism. Second, the goal of this paper was not to produce comprehensive models predicting STIs among adolescents in Malawi; rather, our task was more modest, namely to determine whether adolescent school enrolment, grade attainment, and academic skills are associated with the risk of infection. Thus, information on some factors significantly associated with infection risk was not collected. Third, our measure of adolescent school enrolment status after Round 1 is based on self-report. Some randomized control studies have found a negative association between day-to-day school attendance and STIs (Duflo et al. 2015; Pettifor et al. 2016). Our measures do not take daily attendance into account, which may underestimate the extent to which school enrolment and regular attendance are associated with delay in HSV-2 and HIV infection. Fourth, because our academic skills variables are left-censored, we must restrict the sample for models that include numeracy and literacy and whose origin begins at age 14 to respondents aged 14 at baseline; with this considerably reduced sample we have limited power to find significant effects in these models. Fifth, there is left-censoring of the sample: 12 per cent of respondents were lost to follow-up between first STI testing and the end of the study. Attrition is a cause of concern if the observed and unobserved factors increasing the risk of attrition also increase the risk of infection, leading us to underestimate the incidence of HIV and HSV-2. None of the observed covariates are significantly associated with the risk of attrition among female respondents. School enrolment, however, is negatively associated with the loss to follow-up for male respondents. The attrition of out-of-school young men may be leading us to underestimate the association between school enrolment and STI incidence for this population. Sixth, the measure of literacy is crude and only assesses the ability to read aloud and not comprehension. Finally, as noted earlier, because self-reports of sexual behaviour and condom use are considered to be flawed (Kelly, Hewett, et al. 2014), and because the reliability of self-reports is significantly associated with school enrolment in our data (Soler-Hampejsek et al. 2013), we do not investigate how sexual behaviour potentially mediates the effect of school attendance on infection.

## Conclusion

As is the case for other countries in sub-Saharan Africa that have experienced rapid growth in school enrolment following the abolition of primary school fees, there is a ‘learning crisis’ in Malawi (World Bank 2018). Yet, the results presented here from two districts in the Southern region of the country provide evidence that, even in a setting with deficient school quality and poor learning outcomes, the more grades attained, the less likely girls are to become infected with STIs. For boys, grade attainment is significant for HSV-2, although the negative effect is not robust to different model specifications. Furthermore, for boys, grade attainment is not significantly associated with HIV. The cost of unprotected sexual intercourse is somewhat less consequential for young men, as are the expectations of what constitutes acceptable behaviour for male students. Efforts to encourage adolescents, particularly girls, to stay on track and to continue to progress through school in high-prevalence settings in sub-Saharan Africa could well reduce, or at least postpone, acquisition of STIs. Interventions to increase the number of years of schooling obtained are not only consequential for longer-term health; they are also likely to improve the sexual health of young women during adolescence and early adulthood.

## Supplementary Material

Supplementary Material

## Figures and Tables

**Table 1:** Descriptive statistics, Malawi schooling and adolescent survey 2007–13

	Boys	Girls	Total

Number	1,312	1,337	2,649
*HSV-2 status (percentage)*			
Infected at first testing	9.68	16.98	13.36
Seroconversion	6.63	10.25	8.46
Not infected at last testing	72.56	64.47	68.48
Never tested/indeterminate	11.13	8.30	9.70
*HIV status (percentage)*			
Infected at first testing	0.46	3.29	1.89
Seroconversion	0.69	2.39	1.55
Not infected at last testing	89.86	88.03	88.94
Never tested/indeterminate	8.99	6.28	7.63
*Independent variables*^1^			
Enrolled in school, age 14 (percentage)	83.62	84.67	84.16
Highest grade attained, age 14 (mean, (SD))	5.13 (1.57)	5.53 (1.65)	5.33 (1.62)
Enrolled in school, age 16 (percentage)	68.73	53.92	61.14
Highest grade attained, age 16 (mean (SD))	6.17 (1.91)	6.41 (1.96)	6.29 (1.94)
Academic skills, age 14 (limited to respondents aged 14 in 2007 (*N* = 600))			
Numeracy (mean, (SD)), max = 12	7.74 (3.62)	7.79 (3.86)	7.76 (3.74)
Literate in Chichewa (percentage)	70.89	81.49	76.33
Academic skills, age 16			
Numeracy (mean (SD)), max = 12	8.18 (3.44)	8.04 (3.56)	8.11 (3.50)
Literate in Chichewa (percentage)	79.39	82.81	81.11
Mother’s education (percentage)			
Never attended/don’t know	49.40	45.84	47.58
Less than primary	40.14	41.19	40.68
Completed primary	10.46	12.97	11.75
Father’s education (percentage)			
Never attended/don’t know	32.45	33.03	32.75
Less than primary	41.89	37.60	39.69
Completed primary	25.67	29.36	27.56
Mother dead, age 14 (percentage)	14.68	14.44	14.55
Father dead, age 14 (percentage)	25.69	25.27	25.47
Mother dead, age 16 (percentage)	15.71	16.56	16.14
Father dead, age 16 (percentage)	27.73	27.98	27.85
Ethnicity (percentage)			
Chewa	20.33	19.98	20.15
Lomwe	22.56	23.16	22.87
Yao	41.68	41.27	41.47
Other	15.43	15.59	15.51
HSV-2 prevalence in school catchment area, 2010 (mean, (SD))	13.28 (7.16)	13.09 (7.11)	13.18 (7.13)
HIV prevalence in school catchment area, 2010 (mean (SD))	1.71 (2.40)	1.81 (2.44)	1.76 (2.42)

1 The distributions of the independent variables are calculated for respondents with at least one HSV-2 test result.

*Note*: SD refers to the standard deviation.

*Source*: MSAS.

**Table 2:** Interval-censored regression results for young people in Malawi, 2007–13: HSV-2 (assuming HSV-2 negative at age 14)

	Boys			Girls		
	Coefficient	SE		Coefficient	SE	

In school (enrolled)	0.313	0.170	†	−0.004	0.122	
Highest grade attained	−0.090	0.033	**	−0.073	0.027	**
Mother’s education (ref. Never attended/don’t know)						
Less than primary	−0.006	0.157		−0.036	0.120	
Completed primary	0.297	0.242		0.000	0.188	
Father’s education (ref. Never attended/don’t know)						
Less than primary	−0.095	0.166		−0.090	0.130	
Completed primary	0.029	0.197		0.091	0.149	
Ethnic group (ref. Yao)						
Chewa	0.035	0.180		−0.195	0.142	
Lomwe	−0.145	0.066	*	−0.138	0.051	**
Other groups	−0.246	0.209		−0.318	0.162	*
HSV-2 prevalence in school catchment area, 2010	0.015	0.010		0.035	0.007	***
Mother dead	−0.142	0.195		0.068	0.145	
Father dead	0.234	0.151		0.163	0.118	
Intercept	−24.086	1.683	***	−21.560	1.226	***
Scale parameter	7.474	0.560	***	6.770	0.401	***
Log likelihood	−741.89			−1,063.95		
Chi^2^	24.10		_*_	48.67		***
Number	1,166			1,226		
Observations	4,778			5,094		

† *p* < 0.10;

* *p* < 0.05;

** *p* < 0.01;

*** *p* < 0.001.

*Notes:* HSV-2 is a time-varying covariate measured beginning at age 14. SE refers to the standard error. Ref. is the reference category.

*Source:* Authors’ analysis from MSAS data.

**Table 3:** Interval-censored regression results for young people in Malawi, 2007–13, HIV (assuming HIV negative at either age 14 or age 16)

	Boys						Girls					
	Assume HIV negative at age 14			Assume HIV negative at age 16			Assume HIV negative at age 14			Assume HIV negative at age 16		
	Coefficient	SE		Coefficient	SE		Coefficient	SE		Coefficient	SE	

In school (enrolled)	0.025	0.658		−0.099	0.689		−0.036	0.274		−0.512	0.313	
Highest grade attained	−0.046	0.127		−0.063	0.138		−0.151	0.060	*	−0.112	0.060	†
Mother’s education (ref. Never attended/don’t know)												
Less than primary	0.322	0.589		0.387	0.585		0.206	0.263		0.202	0.262	
Completed primary	−0.117	0.898		0.005	0.892		−0.319	0.424		−0.285	0.423	
Father’s education (ref. Never attended/don’t know)												
Less than primary	−0.246	0.695		−0.279	0.692		−0.137	0.307		−0.163	0.308	
Completed primary	0.467	0.674		0.439	0.671		0.611	0.310	*	0.633	0.309	*
Ethnic group (ref. Yao)												
Chewa	0.485	0.733		0.540	0.734		−0.205	0.315		−0.215	0.314	
Lomwe	0.125	0.245		0.133	0.246		−0.152	0.109		−0.149	0.109	
Other groups	0.475	0.770		0.494	0.772		−0.464	0.357		−0.506	0.358	
HIV prevalence in school catchment area, 2010	0.095	0.094		0.101	0.093		0.232	0.039	***	0.230	0.039	***
Mother dead	1.124	0.538	*	1.050	0.545	†	0.346	0.284		0.445	0.282	
Father dead	1.180	0.574	*	1.044	0.580	†	0.277	0.254		0.180	0.260	
Intercept	−28.102	6.863	***	−29.530	6.374	***	−23.027	2.792	***	−23.805	2.503	***
Scale parameter	7.371	2.285	***	7.911	2.122	***	6.702	0.915	***	6.937	0.823	***
Log likelihood	−87.70			−89.44			−333.91			−344.88		
Chi^2^	16.24			15.26			49.77		***	54.66		***
Number	1,194			1,194			1,253			1,253		
Observations	5,232			5,232			5,625			5,625		

† *p* < 0.10;

* *p* < 0.05;

*** *p* < 0.001.

*Notes*: Time-varying covariates are measured beginning at either age 14 or age 16. SE refers to the standard error. Ref. is the reference category.

*Source:* As for Table 2.

**Table 4:** Interval-censored regression results for young people in Malawi, 2007–13: HSV-2 and HIV (models including academic skills)

	HSV-2 (limited to those aged 14 at baseline)				HIV (assume HIV negative at age 16)				
	Boys		Girls		Boys		Girls		
	Coefficient	SE	Coefficient	SE	Coefficient	SE	Coefficient	SE	

In school (enrolled)	0.361	0.475	−0.319	0.282	−0.094	0.693	−0.521	0.312	†
Highest grade attained	0.095	0.02	−0.135	0.089	−0.041	0.169	−0.147	0.085	†
Numerate	−0.067	0.065	0.046	0.055	−0.022	0.095	0.049	0.051	
Literate in Chichewa	−0.042	0.547	0.503	0.437	–^1^	–	−0.234	0.381	
Scale parameter	7.819***		7.996***		7.816***		7.036***		
Log likelihood	−141.62		−234.77		−89.41		−344.39		
Chi^2^	13.66		25.83*		15.31		55.63***		
Number	275		287		1,194		1,253		
Observations	1,157		1,227		5,232		5,625		

† *p* < 0.10;

* *p* < 0.05;

*** *p* < 0.001.

1 This model does not converge when Chichewa literacy is included; therefore, we exclude this variable from this model.

*Notes:* Other variables included but not shown are maternal and paternal education, ethnic group, HSV-2 or HIV prevalence in school catchment area in 2010, mother dead, and father dead. SE refers to the standard error.

*Source:* As for Table 2.

## Appendix: STI testing procedures

After completing the main survey, the interviewer obtained consent for testing. For those respondents giving consent, HIV status was determined via whole blood obtained from finger pricks using ‘EDTA’ capillary tubes. A serial algorithm of HIV testing was used: if respondents tested positive using the Determine® HIV1/2 test (Abbott, Japan), they were retested using the Uni-Gold™ Recombigen® HIV1/2 test (Trinity Biotech, Ireland). Both tests have a very high sensitivity (100 per cent) and specificity (>99 per cent) in clinical evaluations, including a controlled laboratory setting in rural Kenya (Foglia et al. 2004). In Rounds 4 and 5, SD Bioline HIV 1/2 3.0 (Standard Diagnostics, Inc., South Korea) was used as a tiebreaker in cases in which Determine and Uni-Gold tests gave contradictory results. Note that Bioline was removed from the HIV testing algorithm in Round 6 (2013), in accordance with revised Ministry of Health guidelines. In cases where Determine and Uni-Gold produced discordant results, both tests were repeated in tandem.

The HSV-2 specimens were collected via finger prick in the home or at a private location in Microtainers. (Note that in Round 4 we had planned on using dried blood spots to test for HSV-2. However, a validation exercise conducted for a household-based survey in Uganda a month prior to the Malawi fieldwork indicated that dried blood spots were not optimal for HSV-2 testing and that serum collection was preferable (Nsobya et al. 2016). Additional Institutional Review Board (IRB) clearance was necessary for this change to testing procedures, and this was not obtained until after the start of fieldwork. Although we attempted to retrace respondents who had completed the survey but not been offered HSV-2 testing before the IRB approvals, 168 respondents could not be relocated and tested during the time available.) Serum was separated from the whole blood on the same day of collection by centrifugation at 1,600 *g* for ten minutes. Samples were stored at −20 degrees Celsius in Cryovials®, which were transported weekly to the College of Medicine-Johns Hopkins University Research Project laboratory at Queen Elizabeth Central Hospital in Blantyre, Malawi, for testing. The Kalon™ ElIsA HSV-2 antibody test was used. In an evaluation of several assays using African sera, Kalon was found to have a sensitivity of 92.3 per cent and a specificity of 97.7 per cent (van Dyck et al. 2004) although some validation studies have reported lower values for both measures (Delany-Moreltwe et al. 2010) and others have reported higher values for sensitivity (Biraro et al. 2011). An external validation of the laboratory testing for this study was successfully carried out in 2010 and 2013 by Contract Laboratory Services in Johannesburg.

With a few exceptions, respondents who tested positive for HSV-2 in Rounds 4 or Round 5 were not retested in Round 5 or Round 6, respectively. In view of the small number of samples for which Kalon did not produce a definitive result—1.6 per cent of those tested in Round 4 —indeterminate results were not retested with HerpeSe-lect® as had originally been proposed, for reasons of cost. In Rounds 5 and 6, indeterminate specimens were retested twice with Kalon in an effort to resolve the indeterminacy. After specimen collection, all participants were provided with information about HSV-2 detection, symptoms, ‘safe sex’ practices, and treatment options. HSV-2 test results were made available to study participants at centralized health centres proximate to Balaka and Machinga districts. Respondents were given vouchers with identification numbers to receive their test results and reimbursement for travel to the site. In Round 4, 23.2 per cent of the 1,855 participants tested received their result at a clinic. (Note that because of the change in the testing protocol described here, we were unable to give respondents a fixed date for the start of results dissemination. In the desire to reach as many respondents as possible, including those who might not otherwise have travelled to the clinics, we provided results over the phone for some respondents. An additional 22.4 per cent of Round 4 respondents were reported to have obtained their results via phone.) In Round 5, 26.3 per cent of the 1,763 respondents tested returned to the clinic to obtain their results. In Round 6, 28.4 per cent of the 1,575 participants tested received their result at a clinic and, after an adjustment of the dissemination protocol, an additional 5.0 per cent received their results by phone.
