# Supplementary material for: Does schooling protect sexual health? The association between three measures of education and STIs among adolescents in Malawi
Source: Popul Stud (Camb). Author manuscript; Available in PMC 2020 Jul 1. (PMC7162723; doi:10.1080/00324728.2019.1656282)
Supplement: Supplementary Material [file NIHMS1047630-supplement-Supplementary_Material.pdf]

Supplementary Material to: Does schooling protect sexual health? The association between three measures of education and sexually transmitted infections among adolescents in Malawi - Barbara S. Mensch, Monica J. Grant, Erica Soler-Hampejsek, Christine A. Kelly, Satvika Chalasani and Paul C. Hewett. *Population Studies.* 2019.

Summary of results and robustness checks for interval-censored regression models, Malawi Schooling and Adolescent Survey, 2007–13

A. Models with enrollment and attainment: full sample N = 1166 boys and 1226 girls for HSV-2 and N=1194 boys and 1253 girls for HIV (Tables 2 and 3 in paper)

	HSV-2 (assume negative at age 14)				HIV (assume negative at age 14)				HIV (assume negative at age 16)			
	Boys		Girls		Boys		Girls		Boys		Girls	
In school	0.313	†	-0.004	NS	0.025	NS	-0.036	NS	-0.099	NS	-0.512	NS
Highest grade attained	-0.090	**	-0.073	**	-0.046	NS	-0.151	*	-0.063	NS	-0.112	†

† p<0.10, *p<0.05 **p<0.01

B. Models with enrollment or attainment: full sample N = 1166 boys and 1226 girls N=1194 boys and 1253 girls for HIV

	HSV-2 (assume negative at age 14)				HIV (assume negative at age 14)				HIV (assume negative at age 16)			
	Boys		Girls		Boys		Girls		Boys		Girls	
In school (highest grade attained excluded)	0.155	NS	-0.071	NS	-0.085	NS	-0.189	NS	-0.283	NS	-0.715	*
Highest grade attained (in school excluded)	-0.069	*	-0.074	**	-0.043	NS	-0.153	**	-0.075	NS	-0.151	**

*p<0.05 **p<0.01

C. Models with enrollment, attainment and skills: sample limited to those age 14 at baseline for HSV-2; N= 275 boys and 287 girls for HSV-2 and N=1194 boys and 1253 girls for HIV (Table 4 in paper)

	HSV-2 (assume negative at age 14)				HIV (assume negative at age 16)			
	Boys		Girls		Boys		Girls	
In school	0.361	NS	-0.319	NS	-0.094	NS	-0.521	†
Highest grade attained	0.095	NS	-0.135	NS	-0.041	NS	-0.147	†
Numeracy	-0.067	NS	0.046	NS	-0.022	NS	0.049	NS
Chichewa Literacy	-0.042	NS	0.503	NS	N/A		-0.234	NS

† p<0.10 N/A= model would not converge with literacy

D. Models with enrollment and numeracy and literacy (grade attainment excluded): sample limited to those age 14 at baseline for HSV-2; N= 275 boys and 287 girls and N=1194 boys and 1253 girls for HIV

	HSV-2 (assume negative at age 14)				HIV (assume negative at age 16)			
	Boys		Girls		Boys		Girls	
In school	0.525	NS	-0.419	NS	-0.175	NS	-0.647	*
Numeracy	-0.046	NS	-0.001	NS	-0.035	NS	0.003	NS
Chichewa Literacy	0.071	NS	0.393	NS	N/A		-0.366	NS

*p<0.05 N/A=model would not converge with literacy

E. Models with grade attainment and numeracy and literacy (enrollment excluded): sample limited to those age 14 at baseline for HSV-2; N= 275 boys and 287 girls and N=1194 boys and 1253 girls for HIV

	HSV-2 (assume negative at age 14)				HIV (assume negative at age 16)			
	Boys		Girls		Boys		Girls	
Highest grade attained	0.125	NS	-0.158	†	-0.175	NS	-0.181	*
Numeracy	-0.059	NS	0.042	NS	-0.035	NS	0.045	NS
Chichewa Literacy	-0.021	NS	0.500	NS	N/A		-0.240	NS

† p<0.10, *p<0.05 N/A=model would not converge with literacy

F. Models with numeracy <u>or</u> literacy (grade attainment and enrollment excluded): sample limited to those age 14 at baseline for HSV-2; (N= 275 boys and 287 girls and N=1194 boys and 1253 girls for HIV)

	HSV-2 (assume negative at age 14)				HIV (assume negative at age 16)			
	Boys		Girls		Boys		Girls	
Numeracy (Chichewa Literacy excluded)	-0.010	NS	0.0077	NS	-0.044	NS	-0.046	NS
Chichewa Literacy (Numeracy excluded)	0.065	NS	0.238	NS	N/A		-0.510	†

† p<0.10, N/A=model would not converge with literacy

G. Models with numeracy <u>and</u> literacy (grade attainment and enrollment excluded): sample limited to those age 14 at baseline for HSV-2; (N= 275 boys and 287 girls and N=1194 boys and 1253 girls for HIV)

	HSV-2 (assume negative at age 14)				HIV (assume negative at age 16)			
	Boys		Girls		Boys		Girls	
Numeracy	-0.021	NS	-0.018	†	N/A		-0.016	NS
Chichewa Literacy	0.171	NS	0.356	NS	N/A		-0.420	NS

N/A=model would not converge with literacy; boys model with numeracy alone is in F
